# ReCorDE: a framework for identifying drug classes targeting shared vulnerabilities with applications to synergistic drug discovery

**DOI:** 10.3389/fonc.2024.1343091

**Published:** 2024-05-31

**Authors:** August J. John, Emily T. Ghose, Huanyao Gao, Meagan Luck, Dabin Jeong, Krishna R. Kalari, Liewei Wang

**Affiliations:** ^1^ Department of Molecular Pharmacology and Experimental Therapeutics, Mayo Clinic, Rochester, MN, United States; ^2^ Department of Biological Sciences, University of Notre Dame, South Bend, IN, United States; ^3^ Biochemistry Department, Lawrence University, Appleton, WI, United States; ^4^ Department of Quantitative Health Sciences, Mayo Clinic, Rochester, MN, United States

**Keywords:** cancer, pharmacology, bioinformatics, synergy, PARP, Aurora kinase

## Abstract

Cancer is typically treated with combinatorial therapy, and such combinations may be synergistic. However, discovery of these combinations has proven difficult as brute force combinatorial screening approaches are both logistically complex and resource-intensive. Therefore, computational approaches to augment synergistic drug discovery are of interest, but current approaches are limited by their dependencies on combinatorial drug screening training data or molecular profiling data. These dataset dependencies can limit the number and diversity of drugs for which these approaches can make inferences. Herein, we describe a novel computational framework, ReCorDE (Recurrent Correlation of Drugs with Enrichment), that uses publicly-available cell line-derived monotherapy cytotoxicity datasets to identify drug classes targeting shared vulnerabilities across multiple cancer lineages; and we show how these inferences can be used to augment synergistic drug combination discovery. Additionally, we demonstrate in preclinical models that a drug class combination predicted by ReCorDE to target shared vulnerabilities (PARP inhibitors and Aurora kinase inhibitors) exhibits class-class synergy across lineages. ReCorDE functions independently of combinatorial drug screening and molecular profiling data, using only extensive monotherapy cytotoxicity datasets as its input. This allows ReCorDE to make robust inferences for a large, diverse array of drugs. In conclusion, we have described a novel framework for the identification of drug classes targeting shared vulnerabilities using monotherapy cytotoxicity datasets, and we showed how these inferences can be used to aid discovery of novel synergistic drug combinations.

## 1 Introduction

Cancer is usually treated with a combination of drugs rather than a single agent. Treating cancer with a drug combination decreases the likelihood of the tumor acquiring resistance to therapy. It also allows for lower dosing of individual agents, which can reduce toxicity incidence and severity. Such combinations may be synergistic where the total effect of the drugs in combination is greater than the sum of the individual effects of each drug alone. Identifying such synergistic drug combinations has proven difficult. Brute force combinatorial screening approaches to finding such combinations, even with a small number of drugs, are logistically complex and resource-intensive as the number of assays scales quadratically with individual drugs tested, and combinations must be tested at multiple fixed ratios. Computational approaches for predicting synergy are therefore of interest, and numerous synergy prediction algorithms have been published (1). However, most of these approaches are constrained by their dependency on high-cost, small-sized datasets and/or fail to identify synergistic drug pairs that target closely related, complementary pathways. Here we outline a framework, ReCorDE (Recurrent Correlation of Drugs with Enrichment), for identifying drug classes targeting shared vulnerabilities, and we show how these results can be used to find novel synergistic drug combinations.

Supervised learning-based algorithms, such as DeepSynergy and AuDNNsynergy, rely on synergy-labeled combinatorial drug data for training, which is hard to generate at a large scale (2, 3). Existing synergy-labeled datasets tend to be small and only cover a limited selection of tissue types and drug classes (4–7). These training dataset characteristics limit the utility of supervised synergy algorithms as such approaches often struggle with out-of-domain generalization; thus, accuracy may suffer for predictions involving drug classes or tissues not covered in training data (8).

Unsupervised algorithms, such as DrugComboExplorer and SynGeneNet, that are independent of combinatorial drug response training data have also been developed (9, 10). However, most of these algorithms use molecular profiling data (i.e. ‘omics data) as input. Molecular profiling data is expensive to produce, limiting the size of publicly available datasets. Requirements for perturbational molecular profiling data can add further complications as sample size decreases further, and the number of concentrations tested is typically small, which can provide an incomplete picture of drug activity and result in spurious predictions. Furthermore, because algorithms that use molecular profiling data are designed to predict synergy for a specific molecular profile, predicted synergistic drug pairs may only be synergistic in niche scenarios.

Although large-scale combinatorial datasets for drug screening are difficult to come by, drug response datasets for monotherapy have achieved a scale that allows for identification of response trends across hundreds of cancer cell lines and a large diversity of drug classes. Over the last decade, a handful of medium-throughput efforts have been published to characterize different cancer cell lines’ responses to different drugs in monotherapy. The Genomics of Drug Sensitivity in Cancer (GDSC v2), the Cancer Therapeutics Response Portal (CTRP v2), and the PRISM Drug Repurposing datasets are the largest and most up-to-date of these datasets, each containing sensitivities for hundreds of commercially-available cell lines to hundreds of drugs (11–13). The diversity of cell lines and drugs included in these datasets, along with the comprehensive coverage of drug-cell line combinations, makes these datasets advantageous for large-scale drug response prediction. Furthermore, these three datasets have a general redundancy of cell lines and drugs tested, allowing for cross-validation of drug response across datasets, increasing the confidence of observed drug response patterns.

Multiple existing synergy prediction methods have leveraged monotherapy drug response data to augment their predictions, but these approaches typically require additional ‘omics profiling data as input or require optimization on combinatorial drug response data, which imbue these methods with the limitations described above (1).

Here we present a framework for identifying drug classes targeting shared vulnerabilities, Recurrent Correlation of Drugs with Enrichment (ReCorDE), with applications to finding synergistic drug combinations. Importantly, ReCorDE identifies drug class combinations targeting shared vulnerabilities using large-scale, publicly available monotherapy cytotoxicity data and user-provided canonical mechanism of action without any requirement for combinatorial drug screening or molecular profiling data. Independence from these datasets allows ReCorDE to support a large diversity of drug combinations; furthermore, the simplicity of inputs allows for easy customization and interpretability. ReCorDE results can be used to prioritize drugs for synergy testing under the principle that drugs targeting shared vulnerabilities have interrelated mechanisms of action and therefore have a high potential for exhibiting complementary action-driven synergy (14).

ReCorDE takes inspiration from Narayan et al.’s supervised cophenetic distance-based synergy predictor (15). To prioritize targeting of independent vulnerabilities, Narayan’s model penalizes drug combinations with similar monotherapy response patterns; this approach lends specificity to the algorithm as it eliminates additive drug combinations, but it may miss synergistic drug combinations targeting shared vulnerabilities. Similar drug responses between two drugs may imply: the drugs have similar mechanisms of action; the drugs have different targets but act on the same pathway; or the drugs have different mechanisms of action and target distinct pathways, but these pathways are complementary to each other (shared vulnerability). This latter group of associated drug responses indicates a concept of mutually inclusive survival pathways across cancers and have a high potential for synergy.

To identify drug combinations targeting shared vulnerabilities, ReCorDE first identifies drug pairs that are recurrently correlated across independent monotherapy cytotoxicity datasets. ReCorDE then annotates these drug combinations to drug classes based on user-input and uses enrichment to find class combinations overrepresented in the set of recurrently correlated drug pairs, representing both additive and synergistic combinations. The user is then able to leverage these annotations and their domain knowledge to identify class combinations that involve distinct mechanisms of action, removing likely-additive combinations from analysis and leaving a list of drug classes targeting shared vulnerabilities but with distinct mechanisms of action. These drug class combinations can then be further validated for synergy in follow-up studies.

We validated ReCorDE results using publicly-available combinatorial cytotoxicity data, and we experimentally validated a ReCorDE-identified class combination, poly-ADP ribose polymerase inhibitors (PARPi) and Aurora kinase inhibitors (AurKi), which we showed were synergistic in a variety of cell lines from multiple lineages. We performed a *post-hoc* analysis on publicly-available perturbational transcriptomic signatures for these drug classes, which suggested that generation of reactive oxygen species or PKA/C signaling may underlie PARPi-AurKi synergy.

## 2 Materials and equipment

ReCorDE was implemented using R version 4.3.1 (16). The CTRP and GDSC datasets were obtained from their respective websites while the PRISM dataset was obtained from DepMap portal (**Table 1**) (11–13). CMAP-LINCS data was obtained from CLUE.io (17).

**Table 1:** Monotherapy drug response datasets.

Dataset (Acronym)		Cancer Therapeutics Response Portal (CTRP)	Genomics of Drug Sensitivity in Cancer (GDSC)	PRISM Repurposing Dataset (PRISM)
Version		V2	V2, Release 8.4	Secondary
Raw	Cell Lines Screened	860	969	481
	Drugs Screened	545	286	1448
	Unique Cell Line-Drug Combinations	387,130	235,748	624,224
Post-Data Prep	Cell Lines screened	692	829	481
	Drugs Screened	212	143	258
	Unique Cell Line-Drug Combinations	124,976	98,181	115,130
Reference		(13)	(12)	(11)

Name and version of each dataset. Number of cell lines, drugs, and unique cell line-drug combinations tested in raw data and post-data prep, which refers to datasets after deduplication of drug-cell line combinations, removal of hematological cell lines, and removal of drugs unique to a dataset. Note that not every drug was tested in every cell line and vice versa.

22RV1, LNCaP, DU145, PC3, VCAP, MCF7, HCC70, HCC1395, BT474, MDA453 (MDA-MB-453), HCC1937, MDA231 (MDA-MB-231), and T47D cells were acquired from ATCC (Manassas, VA). Penicillin/streptomycin and RPMI1640, L-15, EMEM, and DMEM media were sourced from Gibco (Grand Island, NY). 10% FBS was obtained from Atlanta Biologicals (Flowery Branch, GA). Alisertib, Tozasertib, Talazoparib, Olaparib, and Rucaparib were sourced from MedChemExpress (Monmouth Junction, NJ).

## 3 Methods

### 3.1 ReCorDE

#### 3.1.1 Correlation construction

**Figure 1:**
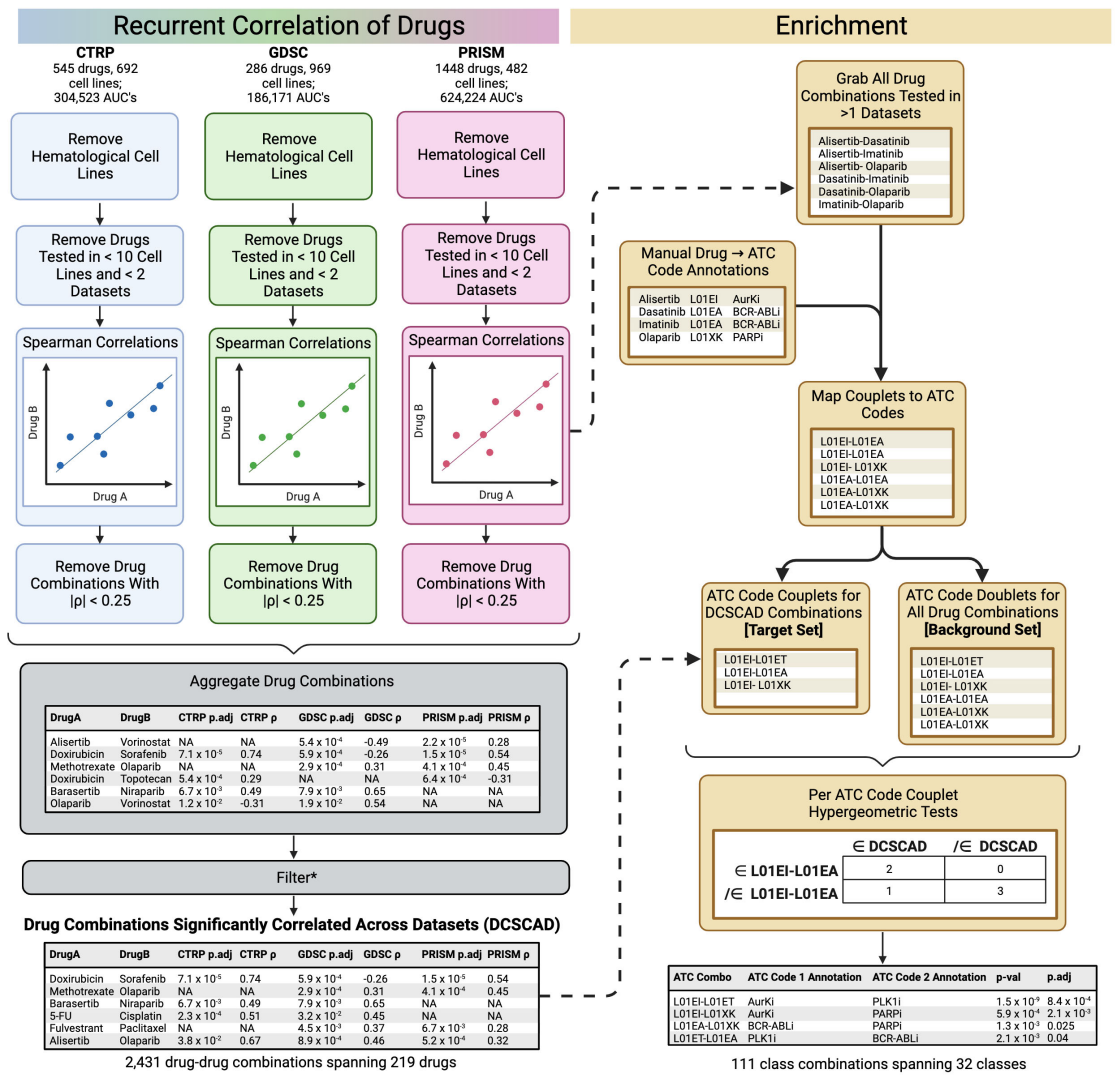
Overview of ReCorDE framework. *Filtering here refers to the removal of drug combinations not significantly correlated in two or more datasets and the removal of drug combinations where the signs for the coefficient of correlation were opposite across datasets.

An overview of the core ReCorDE steps are outlined in **Figure 1**. The code used to implement ReCorDE is available on GitHub (https://github.com/etghose/recorde). To identify drugs targeting shared vulnerabilities, we identified drugs with correlated responses in several datasets. For each dataset, we conducted pairwise Spearman correlations between the AUCs of all possible drug combinations that were tested in at least ten of the same cell lines in two or more datasets (**Supplementary Tables S1**, **S2**). Spearman correlations (18) were implemented using the base R cor.test() function, which uses a Spearman correlation formulation robust to ties. The total number of combinations tested were 22,156 in CTRP, 10,128 in GDSC, and 33,153 in PRISM.

Drug-drug correlations with |ρ| < 0.25 were removed. Drug correlations that met pruning criteria in only two datasets but had opposite signs for their correlation coefficient were also removed. We refer to the remaining set of drug combinations as “drug combinations significantly correlated across datasets” (DCSCAD). P-values were not considered for pruning criteria because they could not be adjusted for multiple testing as they violate exchangeability assumptions due to inter-test variance in sample size and overlap.

#### 3.1.2 Drug class-based enrichment

We used drug class as a surrogate identifier for the mechanism of action. To measure if combinations between drugs of specific classes were enriched in the DCSCAD compared to the distribution of all drug class combinations tested, we assigned each drug to a drug class and, thus, each drug combination to a drug class combination. We then used hypergeometric tests (19) to identify drug class combinations significantly enriched in the DCSCAD compared to the distribution of all drug class combinations tested.

Drugs were assigned to classes based on their Anatomical Therapeutic Chemical (ATC) code (20). Drugs appearing in two or more datasets were manually annotated with their ATC code according to the index provided by the World Health Organization Collaborating Centre for Drug Statistics and Methodology (20). Drugs without an official ATC code were manually assigned to the ATC code best matching the description of their main mechanism of action (**Supplementary Table S2**). We found that a significant number of drugs were annotated to categories encompassing miscellaneous drugs (n=86), often because an official ATC code did not exist corresponding to their drug class or mechanism of action. By lumping dissimilar drugs into the same “miscellaneous” class, the class label is rendered biologically unmeaningful. In the case of two or more “miscellaneous” drugs having the same mechanism of action, we created novel, non-canonical ATC codes and reassigned the previously “miscellaneous” drugs to these newly defined codes. We also altered criteria and/or consolidated certain canonical ATC categories (n=6) to allow closely related drugs to be placed under the same identifier (**Supplementary Methods**). ATC code assignments and explanations of altered canonical and novel non-canonical ATC codes can be found in **Supplementary Tables S2** and **S3**.

We annotated all drug combinations for which Spearman correlations were generated in two or more datasets to a concatenation of the ATC codes (“drug class combinations”) for the individual drugs in a drug combination. For the background distribution, counts for a drug combination, and their corresponding drug class, are proportional to the number of datasets that combination was tested in. For the DCSCAD distribution, counts are proportional to the number of datasets the drug combination is significantly correlated in. This weighting accounts for differences in testing frequency that unduly advantage combinations present in three datasets compared to two datasets. For each drug class combination present in the DCSCAD, we used a hypergeometric test to assess enrichment of that drug class combination in the DCSCAD compared to the background distribution of all drug class combinations tested. We removed drug class combinations in which both drugs belong to the same class before FDR (21) adjustment as these combinations are expected to be enriched and may be less likely to exhibit synergy as they have the same targets. For the final set of enrichment results, we excluded drug class combinations that included individual classes labeled as “miscellaneous-type” classes as these classes are characterized by the drugs’ mechanism of action not fitting into any other classes rather than drugs sharing the same mechanism of action. This property makes these class labels poor descriptors of member drugs’ mechanisms of action. The following classes were considered “miscellaneous”-type classes: D01AE, “other antifungals”; L01AX, “other alkylating agents”; L01CX, “other plant alkaloids”; L01DC, “other cytotoxic antibiotics”; L01EX, “other kinase inhibitors”; and L01XX “other antineoplastics”.

We used a heatmap to easily visualize enrichment results where each cell represents an adjusted enrichment p-value with the rows and columns corresponding to the two different drug classes which make up the class combination being tested for enrichment. To better organize enrichment results and identify biological patterns, drug classes are hierarchically clustered by adjusted enrichment p-value using Euclidean distance and complete linkage. Analyzing enriched correlation patterns among drug classes can offer valuable insight into their mechanisms of action and facilitate their clinical interpretation.

### 3.2 Validation

#### 3.2.1 Validation on OncoPolyPharmacology dataset

ReCorDE does not currently support lineage-specific analysis, and ReCorDE results are not reported on a drug combination level but rather a class combination level. However, there is a paucity of combinatorial cytotoxicity datasets that report synergy on a class combination level, test multiple drugs in the same class, or test multiple lineages. Given the limitations of the available combinatorial cytotoxicity datasets, we used drug combinations that were both present in the DCSCAD and belonged to class combinations enriched in the DCSCAD as a heuristic for ReCorDE-identified synergistic drug combinations. Drug combinations meeting this criterion but with the added caveat of belonging to classes targeting distinct pathways were considered ReCorDE-identified synergistic drug combinations. With this modification, we validated ReCorDE results using combinatorial drug screening data from Merck’s OncoPolyPharmacology screen (6).

Merck’s OncoPolyPharmacology screen consists of synergy measurements for all pairwise combinations between 38 drugs (n=703 drug combinations) performed on a panel of 39 cancer cell lines from 7 different lineages (6). Each drug combination is tested in a 4 x 4 combination scheme for each cell line. Similar to the synergy cutoff used in DeepSynergy, we defined synergy as having a Loewe synergy score greater than the 90^th^ percentile of all synergy scores considered (>8.61) (3). A drug combination was considered synergistic if the Loewe synergy score exceeded this cutoff in at least one cell line. A class combination was considered synergistic if at least one drug combination belonging to that class combination was considered synergistic. Performance was assessed at the class combination level.

#### 3.2.2 Drug combination and synergy analysis for Aurora kinase and PARP inhibitors

##### 3.2.2.1 Cell culture

22RV1, LNCaP, DU145, PC3, HCC1937, T47D, HCC70, HCC1395, and BT474 cells were cultured in RPMI1640 medium supplemented with 10% FBS and 1% penicillin/streptomycin in 5% CO2 conditions at 37°C. MDA453 and MDA231 were cultured in L-15 medium supplemented with 10% FBS and grown in atmospheric conditions. VCAP cells were cultured in DMEM medium supplemented with 10% FBS and 1% penicillin/streptomycin in 5% CO2 conditions at 37°C . MCF7 cells were cultured in EMEM medium supplemented with 10% FBS and 1% penicillin/streptomycin in 5% CO2 conditions at 37°C.

##### 3.2.2.2 Drug combination screening

To measure the combinatorial efficacy of PARP inhibitors (PARPi) and Aurora kinase inhibitors (AurKi), we used an 8 x 5 drug combination matrix-approach, considering all possible pairwise concentration combinations between a given PARPi and AurKi. Cells were treated sequentially with PARPi and AurKi as has previously been done in AurKi-based synergy studies (**Supplementary Methods**) (22). For all assays, cells were plated in 96-well flat bottom plates at 1500-6000 cells per well with 85 µL of cell line media followed by a 24-hour incubation prior to any drug treatment. All assays were performed in triplicate. We prepared drugs in 1:5 serial dilutions for 8 concentrations of a PARPi and 5 concentrations of an AurKi. Cells were treated with 15 µL PARPi or DMSO vehicle and incubated for 3 days. We then washed cells and treated wells with 85 µL of media plus 15 µL AurKi or DMSO vehicle followed by another 3-day incubation. Viability was then determined via CyQuant (ThermoFisher Cat. C7026) cytotoxicity screening, and toxicity effect was compared to vehicle-treated control. In this manner, we assessed the combinatorial efficacy of Talazoparib and Alisertib in the prostate cancer cell lines 22RV1, LNCaP, PC3, DU145, and VCAP and the breast cancer cell lines MCF7, HCC70, HCC1395, BT474, MDA453, HCC1937, MDA231, and T47D. We also assessed the efficacy of other PARPi-AurKi combinations (Olaparib-Alisertib, Rucaparib-Alisertib, and Talazoparib-Tozasertib) in DU145 cells. The final concentration range of Talazoparib (PARPi) was 8 µM - 0.1024 µM, and the final concentration range of both Olaparib and Rucaparib (both PARPi) was 200 µM - 2.45 nM. The final concentration range of Alisertib (AurKi) was 1.6 µM - 2.56 nM, and the final concentration range of Tozasertib (AurKi) was 8 µM - 12.8 nM. We used GraphPad Prism (v10.0.3) to generate dose-response curves in the form of log(inhibitor) vs. response; these curves were fit using the four parameter variable slope option.

##### 3.2.2.3 Drug synergy analysis

Synergy was assessed through multiple methods including Chou-Talalay combination index, Loewe additivity, and highest single agent (HSA) (23–26). For Loewe additivity and HSA metrics, scores > 10 were considered synergistic. For the Chou-Talalay combination index, synergy was defined at EC75, and scores < 0.3 were considered synergistic. Visualization of synergy was also performed through fixed ratio comparison of drug response relative to the single drug alone (**Supplementary Methods**).

### 3.3 Pathway analysis of Aurora kinase inhibitors and PARP inhibitors

Gene expression data for AurKi and PARPi-treated cells were obtained from the CMAP-LINCS database (17). In brief, CMAP-LINCS derives gene expression signatures for various perturbagens using their L1000 system (17). A cell line is treated with a perturbagen for a set concentration and time. Gene expression for 978 “landmark” mRNAs is measured using ligation-mediated amplification. These values are rescaled using L1000 Invariant Set Scaling based on 80 invariant gene transcripts followed by quantile normalization. Expression values for a further 11,350 genes are inferred via linear regression of landmark gene expression values with weights derived from an independent dataset. Gene expression values are then Z-scored gene-wise relative to median plate gene expression using a robust Z-scoring procedure. Finally, replicate-consensus signatures are generated across biological replicates (“Level 5” signatures). CMAP-LINCS designates a subset of these Level 5 signatures as exemplar signatures based on the greatest transcriptional activity score, which is a metric combining the number of differentially expressed genes and signature concordance.

Level 5 gene expression signatures from the CMAP-LINCS database (CMap2020) were downloaded along with corresponding metadata from Clue (17). We extracted PC3-based exemplar signatures using Aurora kinase inhibitors and PARP inhibitors from AurKi-PARPi combinations in the DCSCAD (“treatment” signatures); a DMSO-treated control exemplar signature was also extracted (“control” signature) (**Supplementary Table S4**). We restricted our analysis to the 978 “landmark genes” (17). Signatures used for analysis were generated by substracting the “control” signature values from “treatment” signatures. The resulting signatures were then used to generate pathway activation Z-scores (excluding “Disease-specific” pathways) using Ingenuity Pathway Analysis (27). Pathways with the absolute pathway activation Z-scores > 1 for all signatures were reported (**Supplementary Table S5**).

## 4 Results

### 4.1 ReCorDE results

#### 4.1.1 Drug combinations significantly correlated across datasets

After removing combinations with |ρ| < 0.25, 7726, 2494, and 2677 drug combinations were considered correlated in the CTRP, GDSC, and PRISM datasets, respectively. 2437 of these drug combinations were correlated in ≥ 2 datasets. Most of the drugs recurrently correlated across datasets agreed in the direction of correlation, but 6 drug combinations were removed from this set as the direction of correlation was not the same across all datasets despite meeting ρ magnitude criteria. The remaining 2431 drug combinations showed consistently positive directions of correlation across datasets. This set is referred to as the set of “drug combinations significantly correlated across datasets” (DCSCAD) (**Supplementary Table S6**).

#### 4.1.2 Class combination enrichment

To identify combinations of drugs with different primary mechanisms of action that were overrepresented in the DCSCAD given the types of drugs tested, we tested for enrichment of different class-class combinations in the DCSCAD using hypergeometric tests. We translated each drug combination tested in two or more datasets into a class combination (using drug class as a surrogate identifier for the mechanism of action) using each drug’s Anatomical Therapeutic Chemical (ATC) code (20). This annotates each drug combination with a class combination identifier based upon their ATC codes (i.e. Palbociclib-Everolimus becomes L01EF-L01EG, referring to CDKi-mTORi). For each class combination present in the DCSCAD, we tested if that class combination was enriched in the DCSCAD compared to its distribution in the drug combinations tested for correlation in two or more datasets.

Overall, drug combinations corresponding to 1767 distinct class-class annotations were tested for correlation in two or more datasets, and 564 of these class combinations were present in the DCSCAD. We tested for enrichment of these 564 class combinations in the DCSCAD using hypergeometric tests. After removing same-class combinations (n=32), this number dropped to 532, which were then FDR-adjusted. After excluding “miscellaneous-type”-class-containing combinations (n=150), 111 class combinations were found to be significantly enriched (p.adj < 0.05) in the DCSCAD (**Table 2**; **Supplementary Table S7**).

**Table 2:** Top 5 drug class combinations enriched in DCSCAD targeting different pathways and excluding combinations containing untargeted therapies.

Class A	Class B	P (raw)	P (FDR)
Aurora kinase inhibitors	PARP inhibitors	1.5 x 10^-16^	4.19x 10^-15^
PLK1 inhibitors	Inhibitors of histone-modifying agents—includes BRD and HDAC inhibitors	2.53x 10^-14^	5.62 x 10^-13^
BCR-ABL inhibitors	EGFR inhibitors	7.30 x 10^-12^	1.08 x 10^-10^
BCR-ABL inhibitors	HER2 inhibitors	3.82 x 10^-11^	5.08 x 10^-10^
Aurora kinase inhibitors	Inhibitors of histone-modifying agents—includes BRD and HDAC inhibitors	1.34 x 10^-9^	1.59 x 10^-8^

Also excludes combinations containing “miscellaneous-type” categories. Rows correspond to different drug class combinations identified by Class A and Class B columns. P (raw) contains the raw p-value from the hypergeometric test used to assess enrichment of each drug class combination in the DCSCAD. P (FDR) corresponds to the FDR-adjusted version of this p-value.

**Figure 2:**
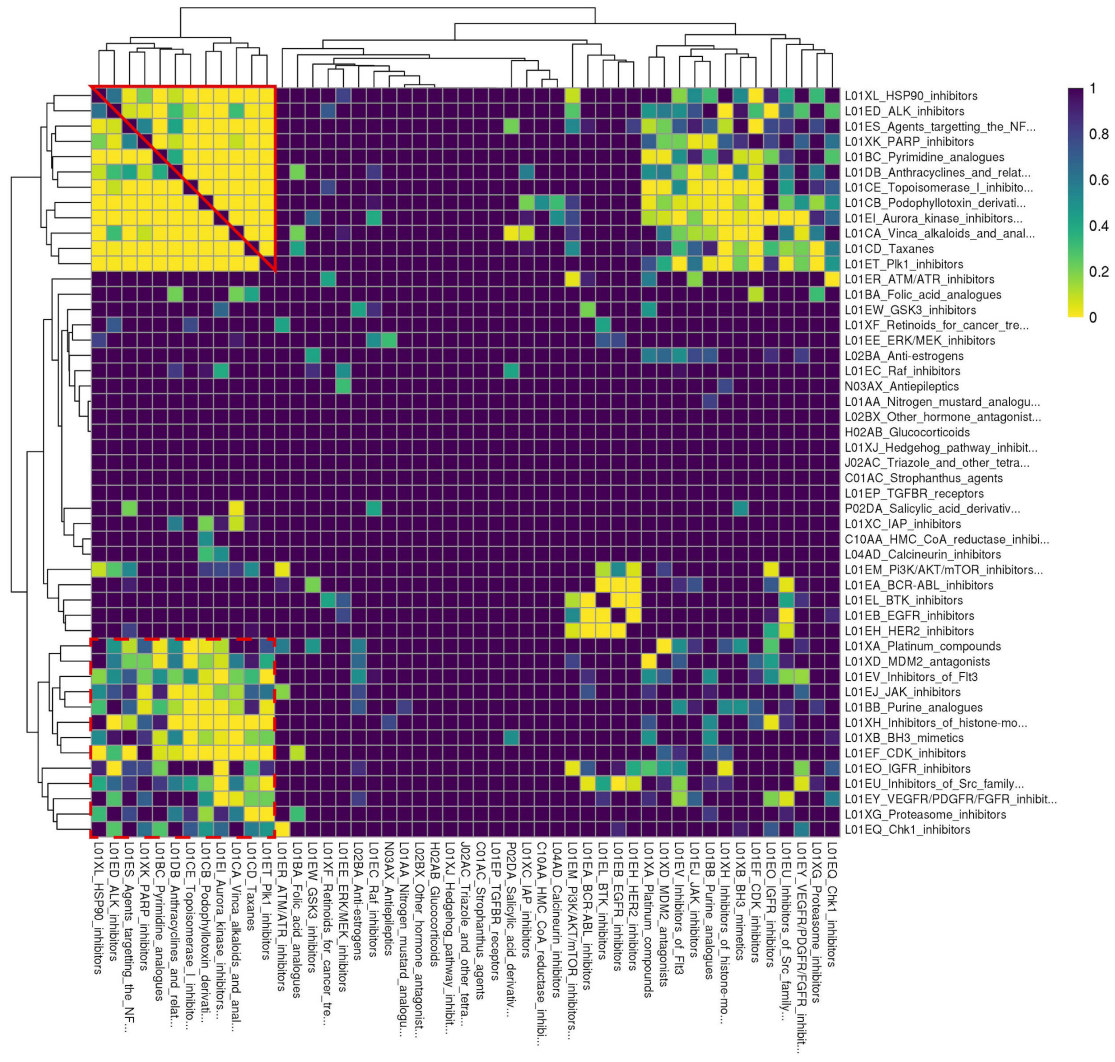
Rows and columns are identical and correspond to different drug classes. Drug classes are labeled with their ATC code followed by a more detailed definition. Each cell corresponds to a drug class combination of the corresponding row and column drug classes. Cell color denotes the FDR-corrected p-value for enrichment of the respective drug class combination in the DCSCAD with yellow corresponding to more significant p-values. This plot was generated based on results after like- and "miscellaneous-type" class removal and after judicious pruning to remove missing cells. Individual classes are hierarchically clustered by FDR-corrected p-value using Euclidean distance and complete linkage. “Cluster A” is outlined in solid red; “Cluster B” is outlined in dashed red.

To facilitate analysis of enrichment results, we converted ReCorDE’s results to a class-by-class square matrix where each entry corresponds to the adjusted p-value for enrichment in the DCSCAD for each class combination. This matrix was visualized with a heatmap (**Figure 2**).

To better organize enrichment results and identify biological patterns in these results, we sought to hierarchically cluster the 54 individual drug classes by adjusted enrichment p-value using complete linkage. However, we found 41 drug class combinations, spanning 17 different individual drug classes, that were not present in the background distribution of drug class annotations, even though individual drugs of each class were present in at least two datasets. The enrichment statistics for these 41 drug class combinations were considered missing values. To avoid removing all 17 individual classes involved in these “missing” class combinations, which would have also removed classes involved in enriched combinations, we chose to remove 5 major offender classes (A02BA, L04AX, L02BB, R05CB, N04CX) to yield a complete square matrix. None of the drugs from these 5 classes were present in drug combinations represented in the DCSCAD, and all of them only contained one drug. This selective pruning approach enabled us to retain as many individual classes as possible in our analysis.

After judiciously pruning 5 individual classes to eliminate class combinations with missing values, we were left with 49 individual classes corresponding to 1176 class combinations. We performed hierarchical clustering of individual classes based on their adjusted p-values for class combination enrichment (**Figure 2**). We observed that when both classes target the same ontological function (e.g. disruption of spindle fiber function), they tend to be enriched in the DCSCAD. However, we did observe a number of enriched class combinations where the classes are not known to target the same pathway or converge on the same ontological function (e.g. HSP90i-TOP1i). The enrichment of these class combinations with distinct mechanisms of action in the DCSCAD suggests that the classes target complementary pathways and therefore have high potential for an underlying synergistic relationship.

**Figure 3:**
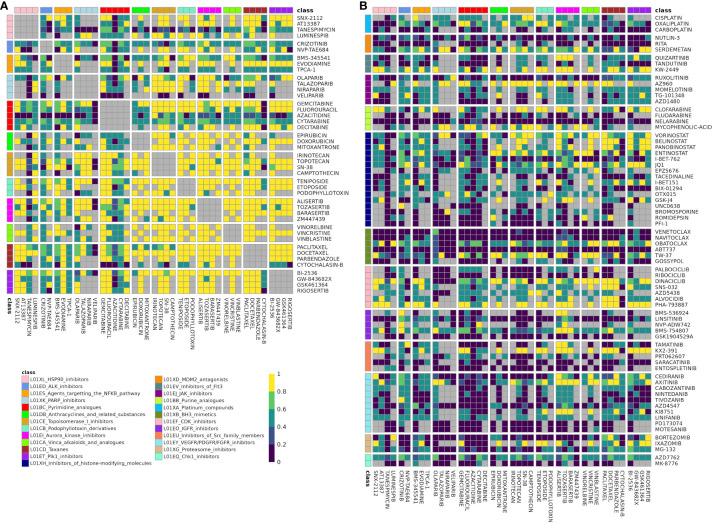
Fraction of datasets with correlation for drugs in class combination clusters-of-interest. Rows and columns correspond to individual drugs in cluster A **(A)** and cluster B **(B)** Drugs are annotated with their class, and cells are split by class-class combination. Cells color denotes the fraction of datasets in which a drug pair was correlated in (|ρ| > 0.25) out of datasets that the drug pair was tested for correlation in. Gray corresponds to like-class drug combinations and to combinations not tested for correlation in any dataset (“missing”). Drug combinations with fraction correlated > 0.66 were included in the DCSCAD.

Hierarchical clustering revealed two clusters-of-interest termed A and B (**Figure 2**) where classes exhibited very similar enrichment patterns. Examining these clusters at the drug level (**Figure 3**) reveals which individual drug combinations drive class combination enrichment and can help the user select which individual agents to use for *in vitro* follow-up testing. Furthermore, visualizing these clusters at the drug level sheds light on intraclass variation in enrichment patterns, which may provide novel biological insight into mechanisms of action. **Figure 3** reveals that the most significantly enriched class combinations are driven by correlations between multiple drugs in each class rather than a single drug, suggesting that ReCorDE is picking up on broad class-class targeting of shared vulnerabilities. Additionally, **Figure 3** shows that drug correlation data can be quite sparse as not every drug combination is tested in an adequate number of cell lines across datasets. By aggregating this data by class, ReCorDE can make inferences about relationships between drugs even when individual drug correlation data is sparse.

When choosing drug class combinations for experimental synergy testing, we selected criteria to emphasize class pairs with limited toxicity. We implemented two criteria to prioritize the 111 enriched class combinations for follow up: 1) if the individual classes target distinct pathways; and 2) if the drugs are both targeted therapy versus at least one being chemotherapeutic. Of the 111 enriched class combinations, 71 met criterion [1]; and 24 met criteria [1] and [2]. The most significantly enriched class combination amongst these 24 were PARPi-AurKi drug combinations (Hypergeometric test, p.adj=4.19 x 10^-15^), which we selected for further experimentation (**Supplementary Table S8**). Further details on this process can be found in **Supplementary Methods**. The top class combinations meeting criteria [1] and [2] are shown in **Table 2**.

### 4.2 Validation of ReCorDE results

#### 4.2.1 OncoPolyPharmacology dataset

Of the 703 drug combinations assessed in the OncoPolyPharmacology dataset, 329 were tested for correlation in ReCorDE, 49 appeared in the DCSCAD of ReCorDE, and 17 belonged to class combinations that were enriched in the DCSCAD (n=13 class combinations) (6). Of these 17 drug combinations, 13 belonged to class combinations enriched in the DCSCAD targeting distinct pathways (n = 10 class combinations). Based on these criteria, 9 out of the 10 class combinations with distinct mechanisms of action that were enriched in the DCSCAD were considered synergistic. More detailed results can be found in **Supplementary Table S9**.

#### 4.2.2 *In vitro* synergy validation

**Figure 4:**
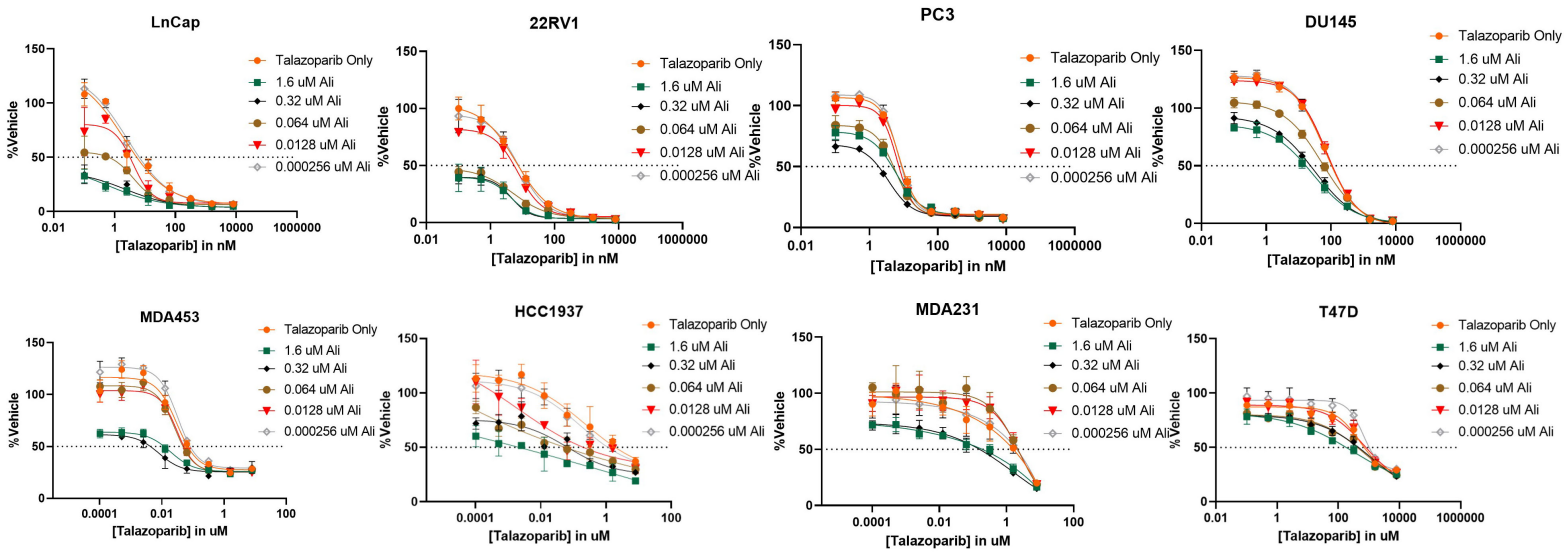
The PARP inhibitor Talazoparib synergizes with the Aurora kinase inhibitor Alisertib (Ali) in prostate cancer cell lines LNCaP, 22Rv1, PC3, and DU145 and breast cancer cell lines MDA453, MDA231, and T47D. An 8-by-5 array of concentrations of Talazoparib-by-Alisertib were tested in triplicate for cytotoxicity via CyQuant reagent. Dose-reponse curves for additional cell lines are available in **Supplementary Figure 2**.

**Figure 5:**
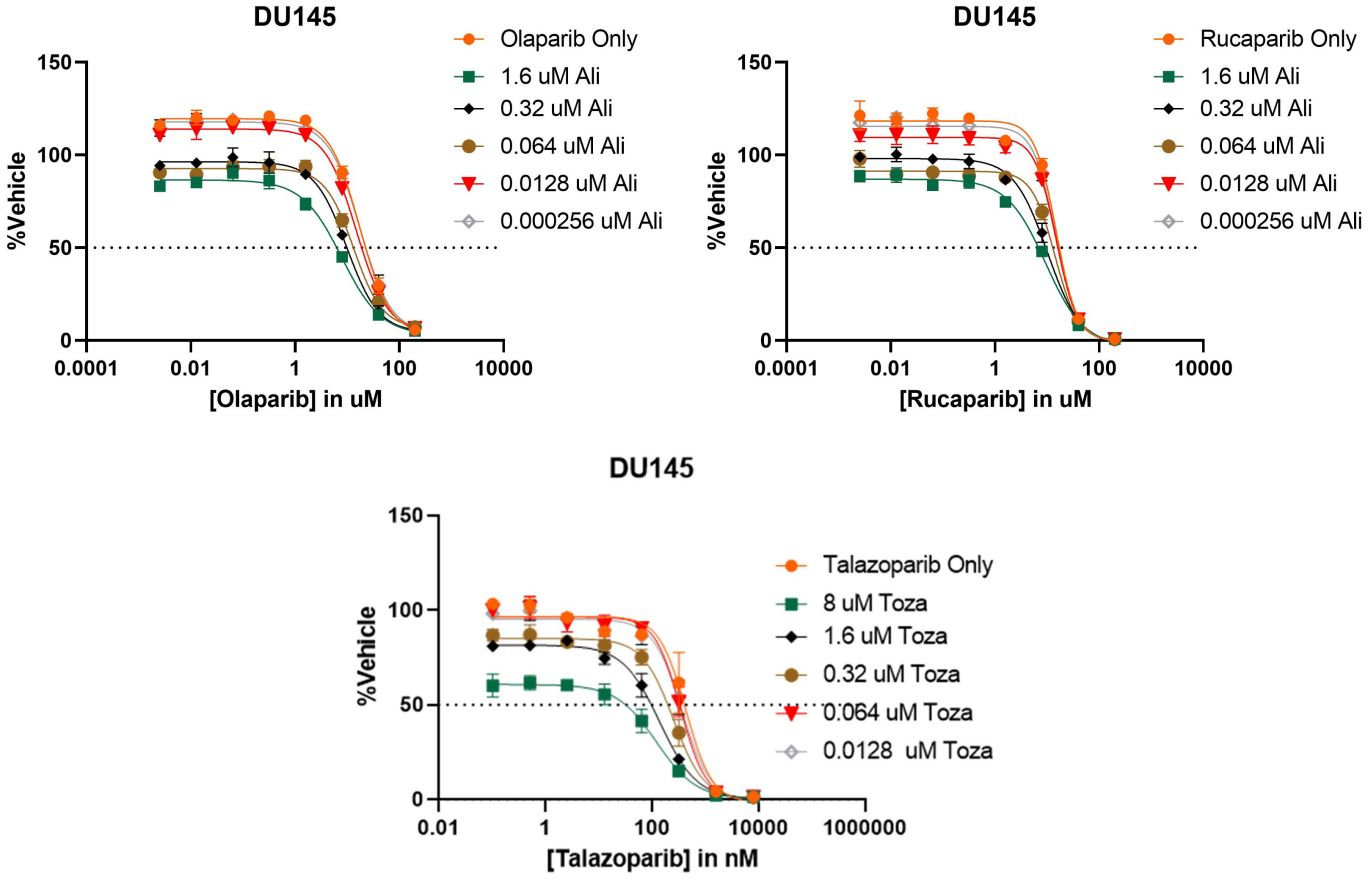
PARP and Aurora kinase inhibitor synergy is observed to be a class-class phenomenon in DU145 cells. (Top) 8 concentrations of two different PARP inhibitors (Olaparib or Rucaparib) were tested in combination with 5 concentrations of the Aurora kinase inhibitor Alisertib (Ali). (Bottom) 8 concentrations of the PARP inhibitor Talazoparib were tested in combination with 5 concentrations of the Aurora kinase inhibitor Tozasertib (Toza). All conditions were tested in triplicate.

After determining that PARP inhibitors and Aurora kinase inhibitors were recurrently correlated in their monotherapy cytotoxic response across non-hematological cell lines, we next sought to determine the synergistic potential of these two agents in combination with one another in a variety of prostate and breast cancer cell lines. Using a drug combination matrix approach, we observed a high degree of synergy between Talazoparib (PARPi) and Alisertib (AurKi) by multiple metrics across prostate and breast cancer cell lines with a higher proportion of prostate cancer cell lines demonstrating the combinatorial phenotype (**Figure 4**, **Table 3**, **Supplementary Figures 1**–**4**). We also observed synergism between other combinations of PARPi and AurKi agents (**Figure 5**). Together, this data demonstrates a robust class-class synergistic relationship between PARP and Aurora kinase inhibitors.

**Table 3:** Summary of Talazoparib and Alisertib synergy screens in prostate and breast cancer cell lines.

Tissue	Cell Line	Chou-Talalay CI	Loewe	HSA	Synergistic Concentrations of PARPi	Synergistic Concentrations of AurKi	BRCA Status
Prostate	22RV1	0.249	14.12	16.43	2.6 – 64 nM	64 – 1600 nM	BRCA2 fs
	LNCaP	0.147	15.15	17.94	0.5 – 12.8 nM	64 – 1600 nM	BRCA2 fs
	PC3	0.198	11.70	10.49	0.5 – 12.8 nM	64 – 1600 nM	BRCA1/2 WT
	DU145	0.267	12.59	14.74	12.8 – 320 nM	64 – 1600 nM	BRCA1 ms BRCA2 ms
	VCAP	1.56104	10.66	4.58	320 – 8000nM	12.8 – 320 nM	BRCA1/2 WT
**Breast**	HCC1395	0.109	11.02	9.37	0.5 – 12.8 nM	64 – 1600 nM	BRCA1 ns BRCA2 ns
	HCC1937	0.097	17.54	33.41	12.8 – 320 nM	64 – 1600 nM	BRCA1 fs
	MCF7	789.019	0.39	5.11	2.6 – 64 nM	64 – 1600 nM	BRCA1/2 WT
	T47D	0.106	12.79	11.93	0.5 – 12.8 nM	64 – 1600 nM	BRCA1/2 WT
	HCC70	0.082	16.10	9.54	0.1 – 2.56 nM	64 – 1600 nM	BRCA1/2 WT
	BT474	Inf	0.26	0.15	64 – 1600 nM	64 – 1600 nM	BRCA2 ns
	MDA-231	26.8825	13.77	10.63	2.6 – 64 nM	64 – 1600 nM	BRCA1/2 WT
	MDA-453	0.91	31.78	8.97	2.6 – 64 nM	64 – 1600 nM	BRCA1/2 WT

Synergy metrics representing response of Talazoparib (PARPi) and Alisertib (AurKi) in individual cell lines. For each cell line, exact synergy metrics by Chou-Talalay combination index (CI), Loewe additivity, and HSA are shown. Individual drug concentrations for highest degree of synergy by Loewe additivity is also provided. Tissue and BRCA status are also indicated for each cell line (28). For BRCA status, WT refers to wild-type, fs refers to frame-shift, ms refers to missense, and ns refers to nonsense. Cell lines where the combination of Talazoparib and Alisertib was deemed synergistic by all three metrics are highlighted in red.

### 4.3 Pathway analysis of PARPi and AurKi agents

We performed an exploratory *post-hoc* analysis of AurKi- and PARPi- gene signatures to investigate possible mechanisms underlying the synergy between these agents. Using drug perturbational gene signatures for AurKi and PARPi, we looked for pathways that were regulated in the same direction by both drug classes in monotherapy. For AurKi and PARPi drugs which were correlated together in the DCSCAD, we pulled available exemplar drug perturbational gene expression signatures done in PC3 cell lines from CMAP-LINCS (PARPi: Olaparib; AurKi: Tozasertib, Barasertib, ZM447439, Alisertib) (17). We then used Ingenuity Pathway Analysis’ activation z-score algorithm on these signatures to identify pathways activated/repressed by each drug class (27). We restricted our analysis to IPA canonical pathways, from which we excluded “disease-specific” pathways to increase the interpretability and relevance of findings (n=313 pathways). We considered pathways where the magnitude of activation z-scores were > 1 and in the same direction for all drugs assessed to be pathways regulated in the same direction by both classes. Two pathways met this criteria for both classes: alpha-adrenergic signaling and NRF2-mediated Oxidative Stress Response (**Supplementary Table S5**). This indicates that PARP and Aurora kinase inhibitors could potentially have a common downstream impact through either oxidative stress or PKA/C signaling.

## 5 Discussion

We have shown that ReCorDE is able to identify drug classes targeting shared vulnerabilities using widely available monotherapy cytotoxicity datasets; and we have shown that this output can be used to identify existing and novel synergistic drug combinations.

The ReCorDE approach has important strengths such as independence from molecular profiling and combinatorial training data. These features, combined with the use of large publicly available monotherapy cytotoxicity datasets, allow ReCorDE to make inferences about targeting of shared vulnerabilities for a large number and diversity of drugs. Furthermore, the use of multi-lineage data allows for identification of robust drug class relationships, applicable to multiple cancer types. Additionally, ReCorDE’s rigorous approach to finding significantly correlated drug pairs increases the accuracy of its inferences. The modular design of ReCorDE also allows for facile expansion of input data without requiring extensive integration efforts. Furthermore, ReCorDE’s simple design allows for straightforward implementation and makes results easily interpretable. Finally, by using a phenotype-driven framework to define drug relationships, ReCorDE is able to prioritize the identification of shared vulnerabilities across cancers that have high translational potential.

While ReCorDE has many strengths, it does have some notable weaknesses such as lack of lineage specificity and requiring manual annotation and disentanglement of redundant targeting.

By not taking lineage information into consideration, ReCorDE has reduced sensitivity for lineage-specific drug class relationships; conversely, this makes ReCorDE inferences broadly applicable to multiple lineages, potentially having greater clinical impact. However, the lack of lineage specificity can make applying ReCorDE to synergistic drug discovery more difficult as it does not explicitly provide information to aid model selection for *in vitro* follow-up. Theoretically, the ReCorDE framework could be extended to make inferences about lineage-specific drug relationships by using linear regression with lineage covariates rather than Spearman correlations, but using this strategy would miss non-linear drug relationships and increase ReCorDE’s sensitivity to outliers; therefore, we have elected to forgo incorporating lineage information into ReCorDE in favor of increased stability.

Additionally, ReCorDE as presented requires manual curation of annotations—this can admittedly be labor-intensive to do manually. If a user wishes to use ATC-based drug classes for annotations as we have, there are a number of automated stand-alone methods for this task that can be used; however, we refrained from using these in our implementation of ReCorDE as these methods can fail to support compounds that are not currently used clinically or may be dependent on perturbational molecular profiling data (29, 30).

Another limitation of our ReCorDE framework is its inability to fully capture the complexities associated with the concurrent application of immunotherapy, anti-angiogenic, and chemotherapy agents, a common scenario in the multifaceted field of cancer therapy. Although our model includes specific angiogenesis-targeted drugs, our cytotoxicity datasets lack vascular and immune contexts, failing to fully delineate the actions of these drug classes. To address this gap, there is a need for comprehensive drug screening datasets, ideally derived from *in vivo* studies or advanced co-culture systems, which could more accurately reflect the complex physiological interactions. Our methodology is flexible and can be updated to include such datasets. We recognize this as an essential area for future enhancement and aim to refine our framework to better accommodate these intricate interactions, thereby improving its clinical applicability and relevance.

Using ReCorDE, we identified prominent patterns of mutual susceptibilities to DNA damaging agents and cell cycle inhibitors. Of particular interest was the identification of shared vulnerabilities to PARP inhibitors and Aurora kinase inhibitors, which also represented a potential novel synergistic cytotoxic drug combination for breast and prostate cancers. The rationale for why these particular drugs are particularly of note is that (a) these drugs, especially PARP inhibitors, have been studied extensively in these cancers but have not been tested in these cancers together; (b) PARP inhibitors are known to be effective in homologous recombination (HR) deficient settings, but resistance is common; and (c) there is prior evidence to suggest connections between the pathways these drugs target. Specifically, DNA damage can have roles in dysregulating mitosis; both HR and Aurora kinase members have been identified as being involved in the DNA damage response, and both Aurora kinase and HR members have been shown to directly be involved in late cell cycle and/or mitosis (31–36). Previous work in two ovarian cancer cell lines has shown Rucaparib and Olaparib synergizing with Alisertib through a proposed Aurora A-induced BRCAness phenotype (37, 38). Additionally, *post-hoc* analysis suggested that the underlying synergy mechanism between these two classes may be through shared perturbation of the NRF2-mediated oxidative stress response or PKA/C signaling. These pathways are known to cross-talk with each other, and the NRF2 pathway has been shown to play a role in the DNA damage response, in which targets of both drug classes play a role (39–42).

## Data availability statement

The original contributions presented in the study are included in the article/**Supplementary Material**. Further inquiries can be directed to the corresponding author.

## Ethics statement

Ethical approval was not required for the studies on humans in accordance with the local legislation and institutional requirements because only commercially available established cell lines were used. Ethical approval was not required for the studies on animals in accordance with the local legislation and institutional requirements because only commercially available established cell lines were used.

## Author contributions

AJ: Conceptualization, Investigation, Validation, Visualization, Writing – original draft. EG: Methodology, Software, Validation, Visualization, Writing – original draft. HG: Conceptualization, Methodology, Writing – review & editing. ML: Investigation, Validation, Writing – review & editing. DJ: Investigation, Validation, Writing – review & editing. KK: Supervision, Writing – review & editing. LW: Supervision, Writing – review & editing.

## Supplementary material

Supplementary Figure S1 Sequential dosing of the PARPi Talazoparib followed by the AurKi Alisertib demonstrates a superior synergistic effect compared to concurrent dosing in 22RV1 cells. 5 concentrations of Talazoparib and Alisertib were tested in triplicate for cytotoxicity via CyQuant Reagent. The concentration ratio of Talazoparib to Alisertib was fixed at 1:25. Chou-Talalay combination index (CI), Loewe additivity, and HSA synergy metrics for each dosing scheme are noted on each panel. (Left) Sequential administration of Alisertib followed by Talazoparib. (Center) Concurrent administration of Alisertib and Talazoparib. (Right) Sequential administration of Talazoparib followed by Alisertib.

Supplementary Figure S2 Additional dose-response curves between Talazoparib and Alisertib. Dose-response curves correspond to assays documented in Table 3 that are not already presented in Figure 4. Assays were performed in the prostate cancer cell line VCAP and the breast cancer cell lines MCF7, HCC70, HCC1395, and BT474.

Supplementary Figure S3 Surface plots depicting Loewe additivity and HSA synergy scores for Talazoparib and Alisertib in the prostate cancer cell lines 22RV1, PC3, LNCaP, and DU145. Most synergistic area scores (MSAS) are noted in the bottom right corner of each plot.

Supplementary Figure S4 Combination index plots for Talazoparib and Alisertib in the prostate cancer cell lines 22RV1, PC3, LNCaP, and DU145. Chou-Talalay combination index (CI) scores are shown for different Talazoparib:Alisertib ratios and ED-cutoffs.

Supplementary Table S1 Name standardization across CTRP, GDSC, and PRISM datasets.

Supplementary Table S2 List of agents present in two or more of the three monotherapy cytotoxicity datasets and corresponding ATC codes.

Supplementary Table S3 ATC code definitions used in ReCorDE.

Supplementary Table S4 CMAP signatures used for post-hoc investigation of PARPi-AurKi synergy..

Supplementary Table S5 Step-by-step results from post-hoc investigation of PARPi-AurKi synergy.

Supplementary Table S6 Drug combinations significantly correlated across datasets (DCSCAD).

Supplementary Table S7 Enrichment results.

Supplementary Table S8 Annotated significant enrichment results.

Supplementary Table S9 ReCorDE validation on Merck’s OncoPolyPharmacology screen.
