# Supplementary figures and images for: ReCorDE: a framework for identifying drug classes targeting shared vulnerabilities with applications to synergistic drug discovery
Source: Front Oncol. 2024 May 31;14:1343091. doi: 10.3389/fonc.2024.1343091 (PMC11176476; doi:10.3389/fonc.2024.1343091)

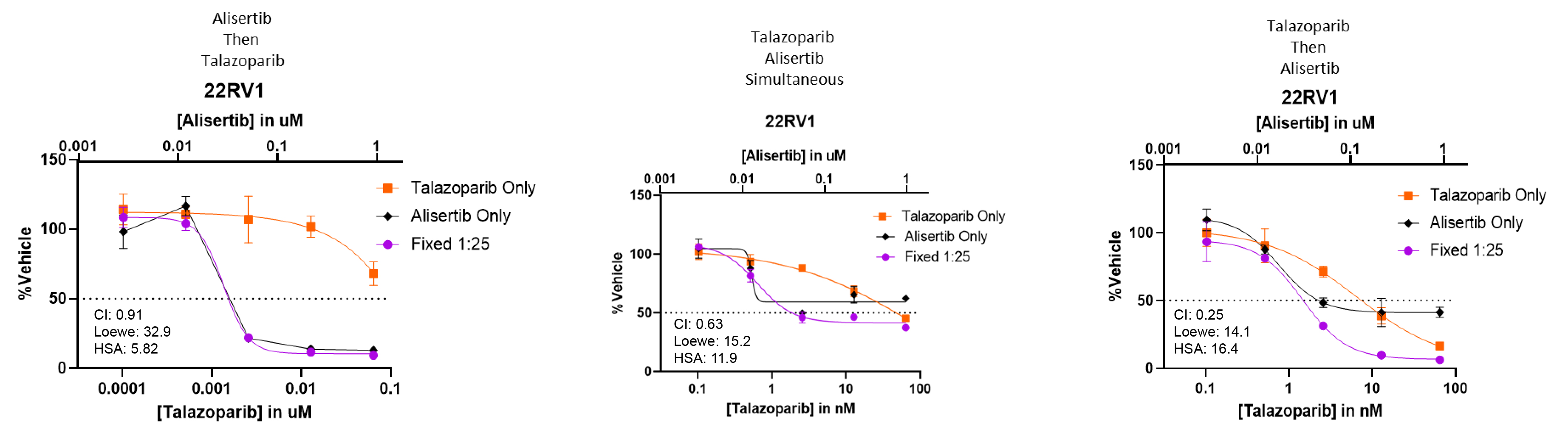

Supplement: Supplementary Figure S1 — Sequential dosing of the PARPi Talazoparib followed by the AurKi Alisertib demonstrates a superior synergistic effect compared to concurrent dosing in 22RV1 cells. 5 concentrations of Talazoparib and Alisertib were tested in triplicate for cytotoxicity via CyQuant Reagent. The concentration ratio of Talazoparib to Alisertib was fixed at 1:25. Chou-Talalay combination index (CI), Loewe additivity, and HSA synergy metrics for each dosing scheme are noted on each panel. (Left) Sequential administration of Alisertib followed by Talazoparib. (Center) Concurrent administration of Alisertib and Talazoparib. (Right) Sequential administration of Talazoparib followed by Alisertib. [file Image_1.jpg]

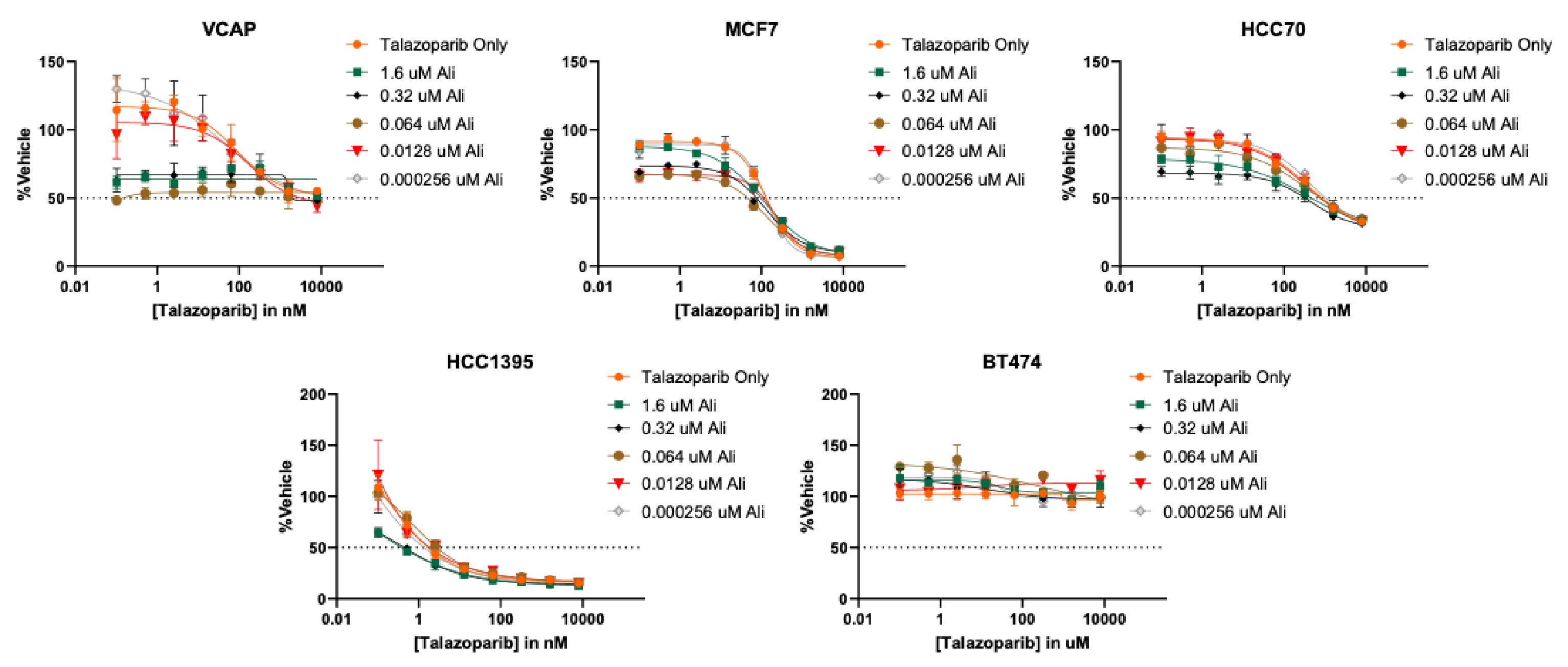

Supplement: Supplementary Figure S2 — Additional dose-response curves between Talazoparib and Alisertib. Dose-response curves correspond to assays documented in Table 3 that are not already presented in Figure 4. Assays were performed in the prostate cancer cell line VCAP and the breast cancer cell lines MCF7, HCC70, HCC1395, and BT474. [file Image_2.jpg]

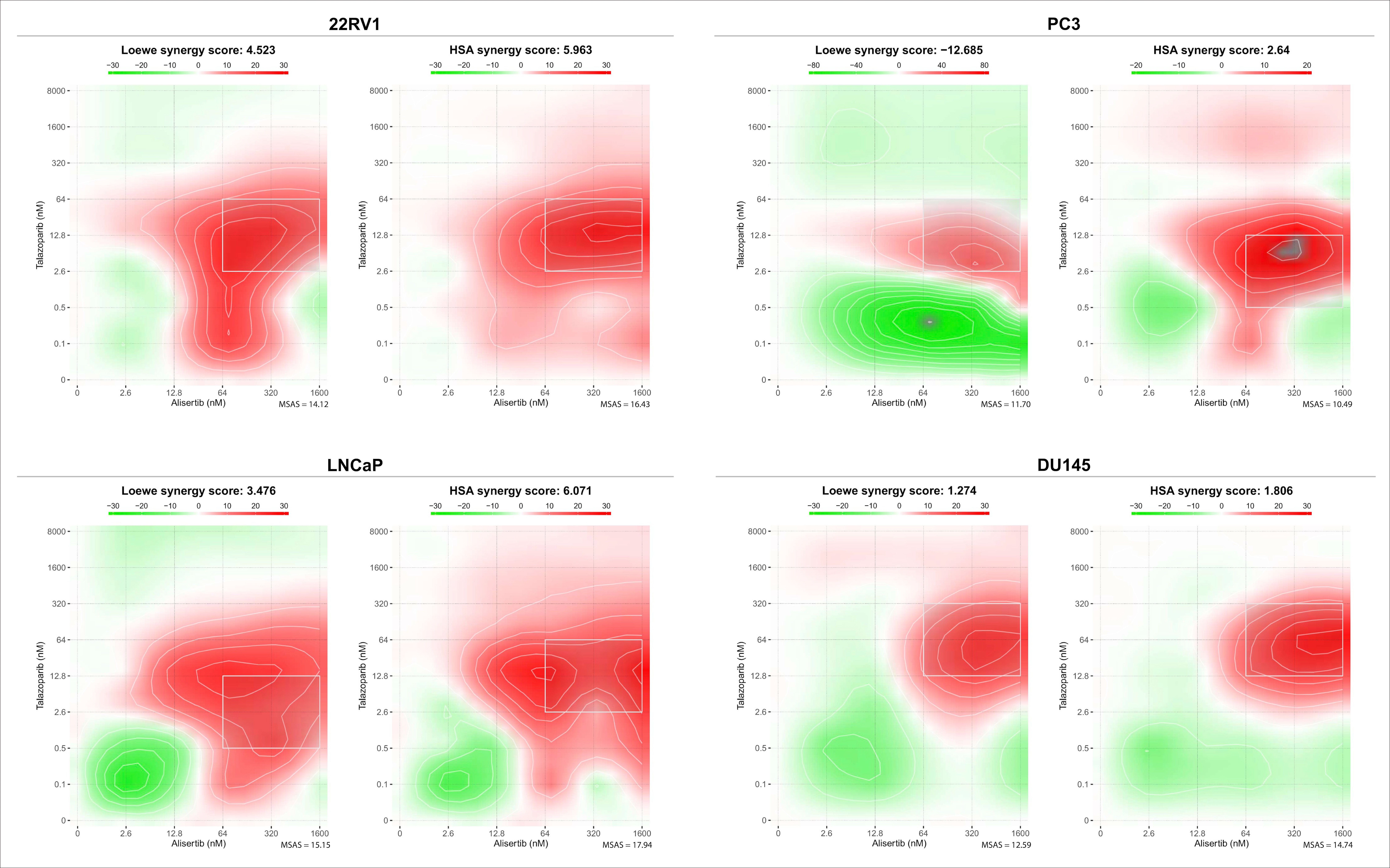

Supplement: Supplementary Figure S3 — Surface plots depicting Loewe additivity and HSA synergy scores for Talazoparib and Alisertib in the prostate cancer cell lines 22RV1, PC3, LNCaP, and DU145. Most synergistic area scores (MSAS) are noted in the bottom right corner of each plot. [file Image_3.jpg]

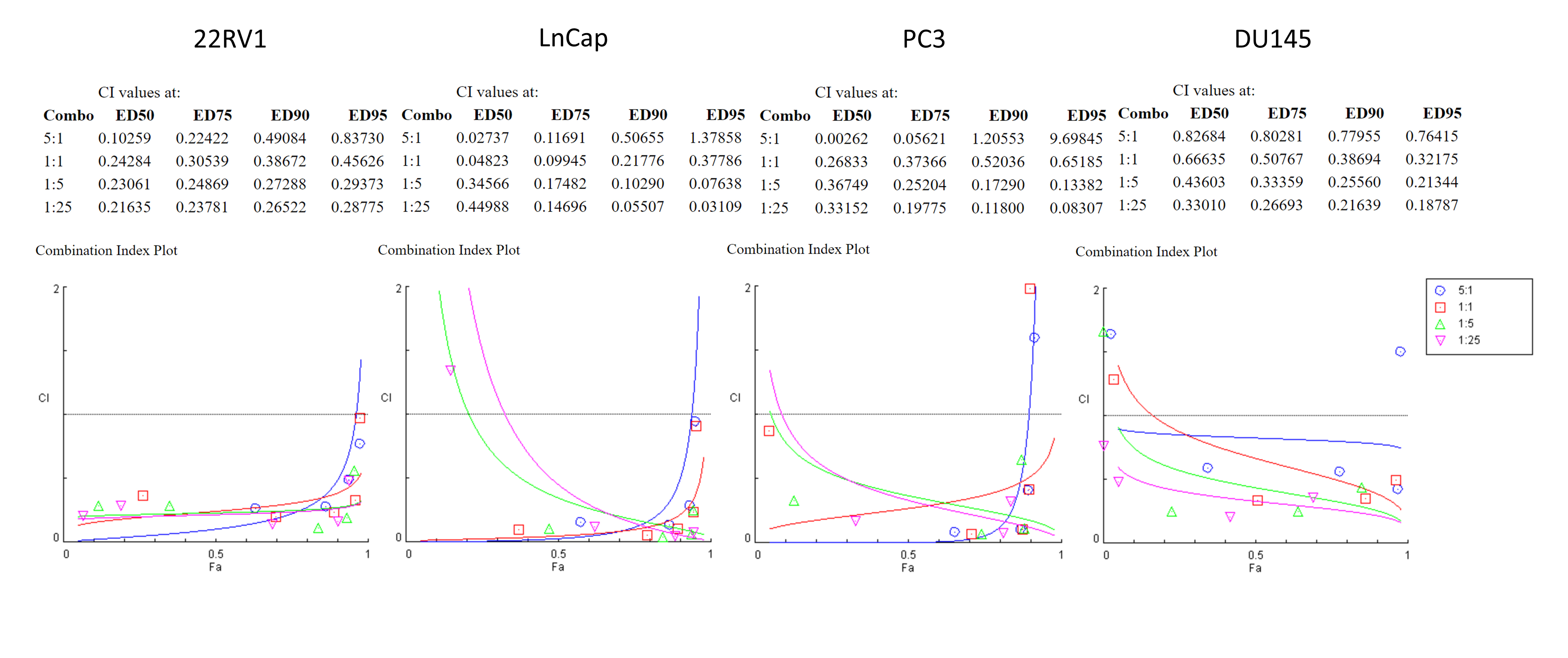

Supplement: Supplementary Figure S4 — Combination index plots for Talazoparib and Alisertib in the prostate cancer cell lines 22RV1, PC3, LNCaP, and DU145. Chou-Talalay combination index (CI) scores are shown for different Talazoparib:Alisertib ratios and ED-cutoffs. [file Image_4.jpg]
