# Supplementary material for: ReCorDE: a framework for identifying drug classes targeting shared vulnerabilities with applications to synergistic drug discovery
Source: Front Oncol. 2024 May 31;14:1343091. doi: 10.3389/fonc.2024.1343091 (PMC11176476; doi:10.3389/fonc.2024.1343091)
Supplement: Supplementary file 6 [file DataSheet_2.pdf]

Supplementary Methods

1 AUC Selection

1.1 GDSC

Duplicate assays for a given cell line-drug combination typically corresponded to assays where the source company for the drug differed between assays or differing concentration ranges were tested. To select a single AUC for a unique cell line-drug combination for duplicate combinations, we prioritized AUC's from assays performed with drug sourced from company ID 1046, the dominant company for drug sourcing by far, and then gave preference to assays with the largest range of concentrations tested. If a cell line-drug combination had multiple assays where the drug, cell line, and source company were identical *and both* were at the maximal concentration range, the assays' AUCs were averaged. Uniquely, Ulixertinib had multiple duplicate assays with identical conditions (same cell line, drug, drug company source, and range of concentrations tested); in this case, AUC's for the assays with matching conditions were averaged to obtain a single Ulixertinib-cell line AUC value.

1.2 PRISM

Duplicate assays for a given cell line-drug combination typically corresponded to assays from different screening runs. When we have duplicate combinations of cell lines and drugs, we opted for a single area under the curve (AUC) value and gave preference to AUCs obtained from assays conducted during the MTS010 screening, as recommended by PRISM dataset guidelines. For drugs-cell line combinations not performed in MTS010, we then gave preference to the AUCs from the HTS002 screening, which was the largest screening intiative. Two drugs, doxycycline and U-0126, each had multiple duplicate assays where conditions and screening runs were the same; in which case, AUC's were averaged.

2 Non-Canonical ATC Code Assignments

86 drugs were initially assigned to "miscellaneous"-type classes as they did not fit cleanly into any pre-existing (canonical) ATC codes. 56/86 of these drugs were placed into 14 completely novel ATC code categories, each defined by their molecular target. The 30 remaining drugs had similar, but not identical targets, to canonical ATC code categories and/or met criteria for multiple ATC code categories. To minimize redundant ATC codes, we removed six canonical ATC codes and replaced them with four noncanonical ATC codes, consolidating categories where drugs frequently hit targets defined by both ATC codes and/or altering criteria to allow inclusion of drugs targeting closely related targets.

L01EE (MEK inhibitors) was altered to include both MEK and ERK inhibitors as there was no canonical ATC code corresponding to ERK inhibitors, and ERK is canonically directly downstream of MEK. L01EG (mTOR inhibitors) and L01EM (Pi3K inhibitors) were consolidated into a single category as multiple drugs were dual inhibitors of both mTOR and Pi3K, and mTOR is downstream of Pi3K; AKT inhibitors were also included in this category as AKT mediates Pi3K activation of mTOR. L01EK (VEGFR inhibitors) and L01EN (FGFR inhibitors) were consolidated

into a single category with altered criteria allowing inclusion of PDGFR inhibitors; these categories were consolidated as drugs frequently inhibit more than one of these receptors. Finally, criteria for L01XH (HDAC inhibitors) was altered to include all histone-modifying agents including BRD inhibitors and histone methylation inhibitors as the substrates of action for all agents are histones.

3 Fixed Ratio Investigation of Dosing Sequence

To investigate the effect of dosing sequence order, we assessed synergy between Talazoparib and Alisertib with concurrent dosing, sequential dosing of Alisertib then Talazoparib, and sequential dosing of Talazoparib then Alisertib. 22RV1 cells were plated in 96-well flat bottom plates at 4000 cells per well with 85 µL of cell line media followed by a 24-hour incubation prior to any drug treatment. All assays were performed in triplicate. We screened 5 concentrations of Talazoparib in combination with 5 concentrations of Alisertib. Concentration ranges were configured such that the concentration ratio of Talazoparib to Alisertib was fixed at 1:25. The final concentration range of Talazoparib was 0.064 µM to 0.1024 nM, and the final concentration range of Alisertib was 1.6 µM – 12.8 nM. For sequential dosing assays, cells were treated with 15 µL of Talazoparib, Alisertib, or DMSO vehicle and incubated for 3 days. We then washed cells and treated wells with 85 µL media plus 15 µL of Talazoparib, Alisertib, or DMSO vehicle followed by another 3-day incubation. Viability was then assessed via CyQuant. For the concurrent dosing assay, cells were treated with DMSO vehicle or 7.5 µL of Talazoparib plus 7.5 µL of Alisertib and incubated for 3 days. Viability was then assessed via CyQuant.

4 Pathway Annotation

For determining if two classes had redundant targeting, enriched class combinations were assigned to four categories: Same Targets, Same Pathways, Similar Pathways, and Different Pathways. Same Targets was used when two or more drugs of each class had a shared target (i.e. EGFR/HER2 therapies). Same Pathway was used when two or more drugs had targets found within the same canonical signaling pathway (i.e. Ras/Raf and MEK/ERK). Similar Pathway was used when two or more drugs from each class had distinct targets in distinct signaling pathways, but those signaling pathways have a similar effect on a downstream effector (i.e. Topoisomerase 1 inhibitors and Platinum compounds). Different Pathways were defined when none of the other classifications of drug signaling were satisfied for two or more agents of distinct classes.
